# Health and Economic Burden of Obesity in Brazil

**DOI:** 10.1371/journal.pone.0068785

**Published:** 2013-07-11

**Authors:** Ketevan Rtveladze, Tim Marsh, Laura Webber, Fanny Kilpi, David Levy, Wolney Conde, Klim McPherson, Martin Brown

**Affiliations:** 1 Micro Health Simulations, London, United Kingdom; 2 Georgetown University, Washington, D. C., United States of America; 3 University of Sao Paulo, Sao Paulo, Brazil; 4 New College, Oxford, United Kingdom; University of Sao Paulo, Brazil

## Abstract

**Introduction:**

Higher and lower-middle income countries are increasingly affected by obesity. Obesity-related diseases are placing a substantial health and economic burden on Brazil. Our aim is to measure the future consequences of these trends on the associated disease burden and health care costs.

**Method:**

A previously developed micro-simulation model is used to project the extent of obesity, obesity-related diseases and associated healthcare costs to 2050. In total, thirteen diseases were considered: coronary heart disease, stroke, hypertension, diabetes, osteoarthritis, and eight cancers. We simulated three hypothetical intervention scenarios: no intervention, 1% and 5% reduction in body mass index (BMI).

**Results:**

In 2010, nearly 57% of the Brazilian male population was overweight or obese (BMI ≥25 kg/m^2^), but the model projects rates as high as 95% by 2050. A slightly less pessimistic picture is predicted for females, increasing from 43% in 2010 to 52% in 2050. Coronary heart disease, stroke, hypertension, cancers, osteoarthritis and diabetes prevalence cases are projected to at least double by 2050, reaching nearly 34,000 cases of hypertension by 2050 (per 100,000). 1% and 5% reduction in mean BMI will save over 800 prevalence cases and nearly 3,000 cases of hypertension by 2050 respectively (per 100,000). The health care costs will double from 2010 ($5.8 billion) in 2050 alone ($10.1 billion). Over 40 years costs will reach $330 billion. However, with effective interventions the costs can be reduced to $302 billion by 1% and to $273 billion by 5% reduction in mean BMI across the population.

**Conclusion:**

Obesity rates are rapidly increasing creating a high burden of disease and associated costs. However, an effective intervention to decrease obesity by just 1% will substantially reduce obesity burden and will have a significant effect on health care expenditure.

## Introduction

### NCDs, Nutrition and Obesity in Brazil

Non-communicable diseases (NCD) are increasingly recognised as the major health issue facing many governments [1]. In 2011, the United Nations (UN) convened the NCD summit to bring together governments to develop a global strategy to confront the problem. Particular attention focused on emerging economies where there has been a rapid increase in the burden of NCDs. A major concern underlying this increased burden is the trend toward increasing obesity [2]. Age-standardised NCD mortality in Brazil (625 per 100,000 people) less than that in Russia, Nigeria, India, and Tanzania (all >700 per 100,000). However, despite its rapid economic growth, Brazil’s NCD mortality is greater than that of the United Kingdom (UK) and Canada (both <400 per 100 000), and almost all other South American countries [3]. Like many nations, Brazil has seen a decline in mortality rates of coronary heart disease (CHD) rates [4] possibly due to improvements in treatment and reduced smoking rates. However, the CHD mortality rates are now expected to rise due to the recent increases in obesity rates [5] along with type-two diabetes those declining rates have been refrained [4]. In addition, Brazil also has one of the fastest aging populations [6] and populations generally gain weight as they age [7] and this could cause a significant challenge for policy makers to tackle burden caused by obesity and obesity in the nearest future.

Brazil has observed a rapid change in the weight profiles of its population, with the prevalence of obesity and overweight increasing partly as a result of the well documented nutrition transition [8], whereby diets move from consumption of fibre and carbohydrate into saturated fats and sugars [9]; [10], as well as increased sedentary behaviour [10]. Monteiro and colleagues (2010) classify three main groups of the food products according to the production processes: unprocessed food - first group; second group - processed food and third - ultra-processed, ready to eat/ready to heat food products which have more saturated fat, sugars, sodium and less fibre comparing to the products from the group 1 and 2 [11]. In Brazil, over the past decades the consumption of the foods from group 1 and group 2 has been replaced by the products from group 3, potentially leading to high rates of obesity and obesity -related diseases [12]. Monterio and colleagues (2010) observed nutrition patterns over two time periods from 1997–1998 and 2002–2003 concluding that consumption of the products from group 3 had been significantly increasing in these two periods whereas use of the items from group 1 and group 2 have declined in turn [11]. The primary reason for a rapid growth in obesity rates is linked to the consumption of products from group 3: the impact of ultra-processed food and drinks causes an obesity epidemic across the world [13]. Surprisingly, the lower consumption of carbohydrates and fat is observed in higher income groups [14] and low income might be a risk factor for unhealthy eating [15].

Brazilian diet has always consisted of high salt and sugar while consumption of green vegetables has been low [12]. However, the consumption of green vegetables and fruit has decreased by 20% over a 16 year time period and in turn, the consumption of biscuits and breads increased by 21% in the same period [13], which is also a source of cheaper calories in the country leading to increased obesity rates [12]; [13]. Moreover, economic transition has a surprisingly adverse effect on health; it is directly linked to increased obesity rates which further exacerbates the economic and disease burden [16]. As a result of nutrition transition, change of diet, increased sedentary life style, associated with increased income, has led to a high obesity rates in Brazil as early as 1975, especially in males [8]. Since 1989 the obesity rate had decreased among higher socio-economic status women in urban areas [17] and was flattening among females in the upper classes in the period 1989–2003 [18].

This is the first attempt to evaluate the burden of obesity and health care cost for Brazil. In this paper, the authors map the trajectory of future weight trends to 2050 based on best currently available data utilising a method already applied in the UK and the US [19] as well as other Latin American countries [20] and Eastern Europe [21]; [29] where obesity and obesity-related diseases have imposed considerable morbidy burden and economic contraints. We measure the future consequences of BMI trends on the associated disease burden and the health care costs of adult Brazilian population (20 years or older). In addition, we estimate the impact of hypothetical interventions aiming on reducing obesity rates by 1% and 5%. There has not been a real life example of such reduction in the world, however, we feel, these goals can be achieved with thorough policy planning and implementation and health and economic benefits will be substantial. The projections are of interest to Brazil as one of the fastest growing economies, but also to other Latin American countries and more generally low and middle income nations that are facing similar issues [21].

## Methods

### BMI and Micro-simulation

A hybrid dataset was created from aggregated BMI data obtained from two nationally representative household health surveys (the 1989 National Survey of Health and Nutrition, and the 2003 World Health Survey) and self-reported telephone survey - VIGITEL for the years 2006 through 2010 [22]; [23]; [24] yielding a total of seven years of data. Adult (20+) BMI distributions were extracted from the surveys by age and gender and three categories of BMI were distinguished: normal weight (≤24.9 kg/m^2^), overweight (25–29.9 kg/m^2^), and obesity (≥30 kg/m^2^) based on the WHO cut-offs [25].

A review of the epidemiological and academic literature was undertaken to determine the country-specific disease incidence, mortality (where applicable), survival or case-fatality rates and annual total medical costs for the following obesity-related 13 diseases: type 2 diabetes, coronary heart disease - CHD (ischemic heart disease (IHD) or myocardial infarction (MI) where CHD data is not available), stroke, hypertension, knee osteoarthritis and 8 obesity-related cancers: breast (female only), kidney, colorectal, oesophageal, endometrial (female), pancreatic, liver and gall bladder by age and sex. Relative risks (RRs) by BMI for each disease were obtained from a systematic review of the epidemiological literature [26]. These RRs are applied, implicitly assuming that the risks to the Brazilian population are reasonably similar to European populations as well as other Latin American countries [20]; [27]?

### Disease Data Collection

For fatal diseases, the micro-simulation requires county specific disease incidence data. Prevalence data can be converted into incidence if necessary using DISMOD-II equations [28]. This converts the prevalence into incidence by dividing the prevalence rate by average duration of the illness, but can only be used for non-terminal diseases. For this project, we have carried out calculations to estimate the incidence of diabetes and hypertension with the following matrix equation where each column independently sums up to 1:

In year k we assigned a person with age a_k_ and gender s and tagged the following probabilities (p): in year k p_0_ (k) represents the probability of being alive without disease d, p_1_(k) indicates the probability of being alive with disease d in year k and p_2_(k) represents the probability of being dead in year k. The probabilities of being alive with (state 0) or without the disease (state 1) and being dead (state 2) have been also assigned to the calculation as well as death statistics p_Ωk (_the probability of dying from disease d in year k (gender s, age a_k_)) and p_ωk_ (the probability of dying from a cause other than disease d in year k (gender s, age a_k_)). The same method has been already utilized for other countries [29].

Although Brazil has a high prevalence of CHD, recent incidence data was not found. Upon consulting experts in the Brazilian government and academic institutions, we used data from 1982 (Table 1) and this may result in overestimating the rates of CHD.

**Table 1:** Disease references used in the micro-simulation.

DISEASE		SOURCE
**CHD**	Incidence	Epidemiology of acute myocardial infarction in Salvador, Brazil: Incidence, Lethality, and Mortality; Lessa I, Cortes E, Souza JA, Filbo JS, Netto JP and Almeida FA; PAHO Bulletin 21(1), 1987
	Mortality	Epidemiology of acute myocardial infarction in Salvador, Brazil: Incidence, Lethality, and Mortality; Lessa I, Cortes E, Souza JA, Filbo JS, Netto JP and Almeida FA; PAHO Bulletin 21(1), 1987
	Survival	Secular Trends in Cardiovascular Disease Mortality, Incidence, and Case Fatality rates in Adults in the United States; Ergin A, Muntner P, Sherwin R, He J, The American Journal of Medicine, 2004∶ 15; Vol. 117, pp. 219–227
	Cost	The burden of hospitalization due to overweight and obesity in Brazil; Sichieri R, Nascimento do S, Coutinho W; Cad. Saúde Pública, Rio de Janeiro, 2007; 23(7): 1721–1727
**Stroke**	Incidence	Trends in stroke incidence, mortality and case fatality rates in Joinville, Brazil: 1995–2006; Cabral NL, Goncalves ARR, Longo AL, Moro CHC, Costa G, Amaral CH, Souza MV, Eluf-Neto J, Fonseca LAM; J Neurol Neurosurg Psychiatry 2009; 80∶ 749–754. doi:10.1136/jnnp.2008.164475
	Mortality	Trends in stroke incidence, mortality and case fatality rates in Joinville, Brazil: 1995–2006; Cabral NL, Goncalves ARR, Longo AL, Moro CHC, Costa G, Amaral CH, Souza MV, Eluf-Neto J, Fonseca LAM; J Neurol Neurosurg Psychiatry 2009; 80∶ 749–754. doi:10.1136/jnnp.2008.164475
	Survival	Stroke Incidence, Prognosis, 30-Day, and 1-Year Case Fatality Rates in Matao, Brazil: A Population-Based Prospective Study; Minelli C; Fu Fen L, Minelli DPC, 2007; Stroke: 30, 2906–2911
	Cost	The burden of hospitalization due to overweight and obesity in Brazil; Sichieri R, Nascimento do S, Coutinho W; Cad. Saúde Pública, Rio de Janeiro, 2007; 23(7): 1721–1727
**Hypertension**	Prevalence	Hypertension Prevalence in the City of Fomiga, MG (Brazil); de Castro RAA, Moncau JEC, Marcopito LF; Arq Bras Cardiol 2007; 88(3), pp.301-206
	Cost	Estimated annual cost of arterial hypertension treatment in Brazil; Dib MW, Riera R and F MB, 2010; Rev Panam Salud Publica 27(2), pp.125–131
**Type 2** **Diabetes**	Prevalence	Prevalence of diabetes and hypertension based on self reported morbidity survey, Brazil 2006; Schmidt MI, Duncan BB, Hoffman JF, de Moura L, Malta DC, de Cavalho RMSV, Rev Saúde Pública 2009; 43(Supl. 2), pp. 1–8
	Cost	The cost of diabetes in Latin America and the Caribbean; Barcelo A, Aedo C, Rajpathak S, Robles S; Bulletin of the World Health Organization; 2003, 81 (1)
**Knee** **Osteoarthritis**	Prevalence	Used the USA figures as the proxy; Please see the methods section.
	Cost	No costs were found.
**All 8 Cancers**	Incidence	Ferlay J, Shin HR, Bray F, Forman D, Mathers C and Parkin DM. GLOBOCAN 2008 v1.2, Cancer Incidence and Mortality Worldwide: IARC CancerBase No. 10 [Internet]. Lyon, France: International Agency for Research on Cancer; 2010. Available from: http://globocan.iarc.fr, accessed on 01/08/2011.
	Mortality	Ferlay J, Shin HR, Bray F, Forman D, Mathers C and Parkin DM. GLOBOCAN 2008 v1.2, Cancer Incidence and Mortality Worldwide: IARC Cancer Base No. 10 [Internet]. Lyon, France: International Agency for Research on Cancer; 2010. Available from: http://globocan.iarc.fr, accessed on 01/08/2011.
	Cost of Breast and Colorectal Cancers	Breakaway: The global burden of cancer - challenges and opportunities; A report from the Economist Intelligence Unit; 2009; http://www.livestrong.org/pdfs/GlobalEconomicImpact
**Cancer** **Survival**	Colorectal	Cancer survival in five continents: a worldwide population-based study (CONCORD). Coleman MP, Quaresma M, Berrino F, et al. Lancet Oncol. 2008 Aug; 9(8): 730–56
	Breast	Five-year survival and prognostic factors in women with breast cancer in Santa Catarina State, Brazil]. Schneider IJ, d’Orsi E. Cad Saude Publica. 2009 Jun; 25 (6): 1285-96. [Article in Portuguese]
	Endometrial	High-risk surgical stage 1 endometrial cancer: analysis of treatment outcome. Viani GA, Patia BF, Pellizzon AC, De Melo MD, Novaes PE, Fogaroli RC, Conte MA, Salvajoli JV. Radiat Oncol. 2006 Aug 3; 1∶ 24
	Other Cancers	Used the USA figures as the proxy; See the methods section.

Cancer incidence and mortality data was acquired from the GLOBOCAN project 2008 [30]. Survival data was obtained only for breast, colorectal and endometrial cancers. For all 8 cancers we have used survival data from the US [19]. By using a proxy country, we probably overestimated the cancer survival since health care expenditure in US is the highest in the world, whereas Brazilian per capita health care spending is nearly 8 times lower than in US [31]. Because data on the incidence of knee osteoarthritis was not found, US data was used as a proxy. Since those rates reflect the obesity distribution of the US, we removed the effect of BMI distribution by extracting the rates of healthy individuals. We then use Brazil’s obesity rates to refine the rates.

We encountered multiple drawbacks during the review process of Brazil-specific cost estimates. We have based our projections on a various scientific articles with different methods of cost calculations. Some of them have only calculated cost of hospitalization (Sichieri 2007 [32]), some have calculated only cost of new cases [33]. All in all, we were able to obtain cost of diabetes [34], CHD [32], stroke [32], hypertension [35], breast cancer and colorectal cancer [33]) for Brazil (Table 1 and Table S2 in File S2). We were unsuccessful in obtaining the costs of the remaining six cancers and knee osteoarthritis therefore these seven diseases have been omitted from cost projections. All the costs obtained were in USD but varied in years; therefore, the costs have been inflated for year 2010 to be uniform during the projections [36].

### Modelling

We employed the two-stage modelling process developed by the UK Foresight working group [37]; [38]; [39]. The software for this model was written in C++. The first module implements a regression analysis based on a series of cross-sectional data using a non-linear multivariate, categorical regression model and is designed to fit to the Brazilian BMI data series by age and sex. The second module implements a micro-simulation programme to produce longitudinal projections by creating a pseudo population cohort based on BMI distributions from module 1. Using a Monte-Carlo simulation method [40], diseases and their associated costs are probabilistically assigned each year as a function of the age, gender and BMI value for the simulated individual. The BMI trajectories are extrapolated using the simulation model with the assumption that an individuals’ BMI percentile in the same age cohort stays the same over time [20]. A simulated cohort member has a risk of getting an obesity-related disease each year if he or she is free of disease at the beginning of the year; the individuals continue living with the disease or die from it depending on disease characteristics (fatal or non- fatal). The progression of the diseases is defined by disease specific survival or case-fatality data, depending on available data. We simulated five million Brazilian individuals by sex and age. Country specific sex and age distributions are based on published projections from the UN Population database by year [41].

After projecting the BMI data forward to 2050, the disease burden associated with this trend has been calculated. The micro simulation enables us to estimate the health burden associated with the projected BMI trends and calculate economic constraints for any hypothetical policy or intervention scenarios in BMI development.

For this project, authors considered to evaluate disease and medical burden for three hypothetical scenarios. Scenario zero assumes no intervention and therefore obesity trends continue as predicted based on the previous pattern (intervention 0). In comparison, the two hypothetical policy scenarios are aiming to reduce the mean BMI by a constant percentage, either one (intervention 1) or 5% (intervention 2) across the whole population. In these two hypothetical interventions, the mean population BMI is reduced by 1% and 5% only in 2010 (a reference year), and the trend is projected from that decreased level forward. A similar method has been applied to Mexico [27] and other 10 countries in the Latin America [20], the Russian Federation [28]), UK and US [19]. Further details of the two part modelling process can also be found in the Lancet and Tackling Obesities Report [19]; [37]; [38]; [39].

No future discounting rate has been applied to calculations since the cost projections are based on increased BMI trends itself and are not directly projected from the reference year cost (2010).

## Results

### BMI

As shown in Table 2 obesity rates are expected to increase for both males and females. In 2010, the model estimates that nearly 57% of the male population is overweight or obese (41% overweight and 16% obese). The rate is predicted to increase to 95% (49% overweight and 46% obese) by 2050. A smaller increase is expected among females, with 2010 rates estimated at 43% (29% overweight and 14% obese) increasing to 52% (32% overweight and 20% obese) by 2050.

**Table 2:** BMI distribution among adult males and females by BMI group (%).

	Males			Females		
Year	BMI ≤24,9 kg/m^2^	BMI 25–30 kg/m^2^	BMI ≥30 kg/m^2^	BMI ≤24,9 kg/m^2^	BMI 25–30 kg/m^2^	BMI ≥30 kg/m^2^
**2010**	43	41	16	57	29	14
**2011**	42	42	16	57	29	14
**2012**	41	42	17	56	29	15
**2013**	39	43	18	56	29	15
**2014**	37	44	19	56	29	15
**2015**	36	44	20	56	29	15
**2016**	34	45	21	56	29	15
**2017**	33	46	21	56	29	15
**2018**	31	46	23	56	29	15
**2019**	30	47	23	55	30	15
**2020**	29	47	24	55	30	15
**2021**	27	48	25	55	30	15
**2022**	26	48	26	54	30	16
**2023**	25	48	27	54	30	16
**2024**	23	49	28	54	30	16
**2025**	22	49	29	54	30	16
**2026**	21	49	30	54	30	16
**2027**	20	50	30	54	30	16
**2028**	18	50	32	53	30	17
**2029**	17	50	33	53	30	17
**2030**	16	50	34	53	30	17
**2031**	16	50	34	52	31	17
**2032**	15	50	35	52	31	17
**2033**	14	50	36	52	31	17
**2034**	13	50	37	52	31	17
**2035**	12	50	38	52	31	17
**2036**	12	50	38	51	31	18
**2037**	11	50	39	51	31	18
**2038**	10	50	40	51	31	18
**2039**	10	50	40	51	31	18
**2040**	9	50	41	51	31	18
**2041**	8	50	42	50	31	19
**2042**	8	50	42	50	31	19
**2043**	7	50	43	50	31	19
**2044**	7	50	43	50	31	19
**2045**	6	50	44	49	32	19
**2046**	6	50	44	49	32	19
**2047**	6	50	44	49	32	19
**2048**	5	50	45	49	32	19
**2049**	5	50	45	48	32	20
**2050**	5	49	46	48	32	20

Similar patterns are observed by age. Among men, the growth is found in all age groups. The increase is most prominent in men aged 25–49, and in older age groups, 70 and above. Generally, similar results are observed among females, but, in some age groups, the prevalence of normal weight is greater than that of obese and overweight. In 2030, the percent of females aged 40–49 that are normal weight is higher than the percent overweight and obese. Among the 30–39 year old age group the distribution varies. By 2030, the distribution among females aged 30–34 remains steady and slightly increases until age 39. After age 49, an increase in the BMI percentage is seen in all age groups (Table 3). By 2050 obesity and overweight overcomes the normal weight 5–6 times in males in every age group, whereas in females, obesity rates are 1–3 times lower than normal weight with exception of age group 55–59 and 60–64 (Table 3). The detailed figures have been presented in File S1 as Figures S1–S24.

**Table 3:** Projected distribution of BMI by age group and BMI group (%).

		2010				2020			2030			2040			2050	
		BMI %														
Male	Age	≤24.9	25–29.9	≥30	≤24.9	25–29.9	≥30	≤24.9	25–29.9	≥30	≤24.9	25–29.9	≥30	≤24.9	25–29.9	≥30
	**20–24**	62	29	8	43	40	17	25	46	29	12	48	40	6	48	46
	**25–29**	48	39	13	30	48	22	16	52	32	8	52	40	4	51	46
	**30–34**	41	43	16	25	49	26	14	51	36	7	50	43	4	49	48
	**35–39**	39	43	18	25	48	27	12	51	35	8	50	42	4	50	46
	**40–44**	37	45	18	25	49	26	16	51	34	9	51	40	5	50	45
	**45–49**	36	44	2	23	49	28	13	51	35	7	51	42	4	50	46
	**50–54**	36	43	2	23	46	30	14	47	39	8	46	46	4	46	50
	**55–59**	36	44	19	24	50	26	14	53	33	8	53	39	4	53	43
	**60–64**	38	44	18	25	48	27	14	50	36	8	49	43	4	48	48
	**65–69**	44	42	14	30	50	20	18	57	25	10	59	31	6	59	36
	**70–74**	44	43	14	30	48	22	18	51	31	10	51	39	6	50	45
	**75+**	53	36	11	37	43	20	22	46	32	12	46	42	6	46	48
		**BMI %**														
**Female**	**Age**	**≤24.9**	**25–29.9**	**≥30**	**≤24.9**	**25–29.9**	**≥30**	**≤24.9**	**25–29.9**	**≥30**	**≤24.9**	**25–29.9**	**≥30**	**≤24.9**	**25–29.9**	**≥30**
	**20–24**	78	16	5	74	19	7	70	22	8	65	25	10	60	28	12
	**25–29**	70	21	9	66	23	11	61	24	14	57	26	18	52	27	22
	**30–34**	63	26	11	62	26	12	62	25	13	61	25	14	60	25	15
	**35–39**	59	28	13	58	24	15	56	26	17	55	25	20	54	24	22
	**40–44**	55	31	15	56	30	14	58	29	14	59	28	13	60	27	13
	**45–49**	50	33	17	52	31	17	54	30	16	56	28	16	58	27	16
	**50–54**	45	36	20	43	38	19	41	40	19	40	42	18	38	44	18
	**55–59**	42	36	22	38	37	25	35	38	27	31	39	30	28	39	33
	**60–64**	41	37	22	36	40	24	32	43	25	28	45	27	25	47	28
	**65–69**	43	37	19	42	39	19	41	40	20	39	41	2	38	42	20
	**70–74**	44	36	20	39	38	22	35	41	25	30	43	27	26	45	29
	**75+**	49	33	18	42	36	22	35	39	26	28	42	30	23	43	34

### Diseases

High obesity rates have resulted in increased disease burden, causing significant increase in morbidity rate for all projected disease. Prevalence of CHD, stroke, 8 cancers, knee osteoarthritis and type 2 diabetes is projected to double between 2010 and 2050. The prevalence rate of hypertension is projected to reach over 34,000 in 2050 cases compared to 21,000 in 2010 (per 100,000). Prevalence of type 2 diabetes nearly doubles from 2010 to 2050 rising up to over 6,000 cases (per 100,000). Knee osteoarthritis is projected to double, whereas CHD, stroke and cancer prevalence cases will triple by 2050 (Table 4). The new cases of diseases also have been projected and can be found in File S3 as Table S3.

**Table 4:** Prevalence cases for all adults (per 100,000). The numbers in square brackets represent confidence intervals.

Intervention 0		8 Cancers	CHD & Stroke	Knee Osteoarthritis	Type 2 Diabetes	Hypertension
***Year***	2010	281 [±5]	1,155 [±10]	12,728 [±32]	3,079 [±16]	21,064 [±41]
	2020	379 [±5]	1,664 [±11]	15,107 [±34]	3,799 [±17]	25,066 [±43]
	2030	463 [±6]	2,109 [±12]	17,755 [±36]	4,557 [±18]	28,824 [±46]
	2040	548 [±6]	2,654 [±14]	20,320 [±38]	5,345 [±20]	31,999 [±48]
	2050	615 [±7]	3,148 [±15]	22,393 [±41]	6,009 [±21]	34,336 [±51]
**Intervention 1**		**8 Cancers**	**CHD & Stroke**	**Knee Osteoarthritis**	**Type 2 Diabetes**	**Hypertension**
***Year***	2010	280 [±5]	1,149 [±10]	12,757 [±32]	3,075 [±16]	21,053 [±41]
	2020	378 [±5]	1,635 [±11]	14,968 [±34]	3,678 [±17]	24,724 [±43]
	2030	455 [±6]	2,072 [±12]	17,484 [±36]	4,338 [±18]	28,206 [±45]
	2040	539 [±6]	2,570 [±14]	19,989 [±38]	4,992 [±19]	31,219 [±48]
	2050	610 [±7]	3,035 [±15]	21,988 [±40]	5,540 [±20]	33,483 [±50]
**Intervention 2**		**8 Cancers**	**CHD & Stroke**	**Knee Osteoarthritis**	**Type 2 Diabetes**	**Hypertension**
***Year***	2010	278 [±5]	1,151 [±10]	12,729 [±32]	3,075 [±16]	21,046 [±41]
	2020	370 [±5]	1,568 [±11]	14,602 [±33]	3,456 [±16]	24,204 [±43]
	2030	451 [±6]	1,951 [±12]	16,977 [±35]	3,951 [±17]	27,222 [±44]
	2040	534 [±6]	2,426 [±13]	19,305 [±37]	4,476 [±18]	29,741 [±46]
	2050	604 [±7]	2,859 [±15]	21,112 [±40]	4,900 [±19]	31,568 [±48]

*Intervention 0– BMI projections according to the past trend; Intervention 1–1% reduction in BMI across the population in 2010;*

*Intervention 2–5% reduction in BMI across the population in 2010.*

A 1% reduction in mean BMI would save over 15,000 prevalence cases in 2030 and nearly 10,000 cases in 2050 for cancers. The figures are even higher for CHD and stroke, the over 72,000 cases in 2030 and over 222,000 in 2050 (for whole Brazilian population). Over 0.7 million cases of knee osteoarthritis can be prevented with 1% reduction in BMI by 2050, whereas the figure for 2030 is over 72,000 in the whole population. Nearly 0.5 million cases of type 2 diabetes can be avoided with this intervention in 2030 and nearly 0.8 million cases in 2050. The avoided prevalence cases are highest for hypertension with over 1.2 million in 2030 and 1.6 million cases in 2050 being avoided in the total population.

The figures are even higher if mean BMI in the population decreased by 5%. The country could save nearly 6,000 cases of cancers in 2030 and over 21,000 in 2050. Over 310,000 and nearly 600,000 cases of CHD and stroke can be avoided in 2030 and 2050 respectively. 1.5 million and 2.5 million of knee osteoarthritis prevalence cases can be saved if BMI is reduced by 5%. Again, high numbers are observed for type 2 diabetes (1.1 million and 2.1 million in 2030 and 2050 respectively). Over 3.1 million cases of hypertension can be prevented in 2030, whereas this number can reach over 5.4 million in 2050 (per population). The figures have been presented per 100,000 of population in Table 5.

**Table 5:** Prevalence cases avoided from year 2010 by interventions (per 100,000 of population in 2010).

Intervention 1					
Year	8 Cancers	CHD & Stroke	Knee Osteoarthritis	Type 2 Diabetes	Hypertension
2010	1 [±7]	6 [±14]	0 [±45]	4 [±22]	11 [±58]
2020	1 [±8]	29 [±16]	139 [±49]	121 [±24]	342 [±63]
2030	8 [±9]	37 [±18]	271 [±53]	219 [±27]	618 [±68]
2040	9 [±9]	84 [±20]	331 [±57]	353 [±29]	780 [±71]
2050	5 [±10]	113 [±22]	405 [±60]	469 [±30]	853 [±74]
**Intervention 2**					
**Year**	**8 Cancers**	**CHD & Stroke**	**Knee Osteoarthritis**	**Type 2 Diabetes**	**Hypertension**
2010	3 [±7]	4 [±14]	0 [±45]	4 [±22]	18 [±58]
2020	9 [±8]	96 [±16]	505 [±49]	343 [±24]	862 [±63]
2030	12 [±9]	158 [±18]	778 [±53]	606 [±26]	1,602 [±67]
2040	14 [±9]	228 [±20]	1,015 [±56]	869 [±28]	2,258 [±70]
2050	11 [±10]	289 [±22]	1,281 [±59]	1,109 [±30]	2,768 [±73]

The numbers in square brackets represent confidence intervals.

*Intervention 0– BMI projections according to the past trend; Intervention 1–1% reduction in BMI across the population in 2010;*

*Intervention 2–5% reduction in BMI across the population in 2010.*

### Costs

In the absence of intervention, obesity and overweight-related disease costs substantially increase between 2010 and 2050, increasing from $160 million to $313 million for female breast cancer in 2010 and 2050 respectively (US Dollars). The colorectal costs are expected to grow from $115 million to $214 million in the same time period. The costs of CHD and stroke more than double from 2010 to 2050 reaching $180 million for CHD in 2050 and $23 million for stroke. Hypertension medical costs are expected to increase from $445 million to $657 million in 2010 and 2050 alone. Diabetes imposes the highest health care expenditure, and it is projected to rise from $5 billion to $8.7 billion alone in 2010 and 2050 respectively. The costs have also been produced by gender and all are presented in Table 6.

**Table 6:** Estimated disease costs according to the hypothetical scenarios.

Intervention 0							
	Cancers		Cardio Vascular Diseases				Total
Year	Breast*	Colorectal	CHD	Stroke	Hypertension	Diabetes	
2010	160	115	81	9	445	5,005	5,815
2015	183	124	97	12	485	5,551	6,452
2020	203	135	110	13	519	5,997	6,977
2025	223	155	123	14	550	6,443	7,508
2030	244	167	134	15	579	6,944	8,083
2035	266	179	146	18	603	7,439	8,651
2040	285	192	157	19	624	7,888	9,165
2045	302	204	171	22	642	8,327	9,667
2050	313	214	180	23	657	8,718	10,106
2010–2050•	9,950	6,753	5,490	667	23,326	284,261	330,447
**Intervention 1**							
	**Cancers**		**Cardio Vascular Diseases**				**Total**
**Year**	**Breast** *	**Colorectal**	**CHD**	**Stroke**	**Hypertension**	**Diabetes**	
2010	160	115	81	9	445	5,005	5,540
2015	181	128	96	12	482	5,480	6,069
2020	203	135	108	13	513	5,866	6,500
2025	223	149	119	14	542	6,276	6,951
2030	245	165	129	15	568	6,713	7,425
2035	266	168	141	18	590	7,129	7,878
2040	283	186	152	19	608	7,517	8,296
2045	298	200	162	22	624	7,874	8,682
2050	310	212	171	23	637	8,169	9,000
2010–2050•	9,907	6,649	5,285	661	22,894	273,964	302,803
**Intervention 2**							
	**Cancers**		**Cardio Vascular Diseases**				**Total**
**Year**	**Breast** *	**Colorectal**	**CHD**	**Stroke**	**Hypertension**	**Diabetes**	
2010	160	115	81	9	445	5,005	5,540
2015	180	132	91	12	475	5,236	5,813
2020	202	150	99	13	501	5,453	6,066
2025	220	143	108	14	523	5,706	6,351
2030	241	156	118	15	542	6,002	6,677
2035	264	187	126	17	556	6,314	7,013
2040	283	197	135	18	566	6,593	7,312
2045	298	207	144	21	572	6,830	7,566
2050	309	216	151	22	575	6,962	7,710
2010–2050•	9,861	6,912	4,812	629	21,755	246,672	273,868

* Females only;

•The numbers to do not sump up since this is a total cost from 2010–2050 whereas other costs represent the actual cost in a particular year.

A 1% reduction in mean BMI across the population will lead to reduction in health care expenditure. The total health care cost with no intervention was projected over $330 billion over 40 years (2010 to 2050). However, a 1% decrease in mean BMI will lead to saving of over $27 billion saving in the same time period, whereas this number will increase to over $56 billion from 2010 to 2050 with 5% decrease in mean BMI. The savings will reach over $10 billion for diabetes over 40 years by a 1% decrease in mean BMI and over $37 billion by a 5% reduction in mean BMI. A substantial reduction is also observed for hypertension, nearly $0.5 billion and $1.6 billion with a 1% and 5% reduction.

### Limitations

Several limitations should be noted. The ability to obtain sex and age specific disease incidence data is limited, requiring the use of prevalence rates. For some calculations, we used proxy data (e.g., the US knee osteoarthritis data was applied to the Brazilian population) and limited accuracy. We have used CHD incidence data from 1982 and perhaps we have overestimated the burden of the disease, since the incidence rates may be declining due to smoking reduction and enhancements in medical treatment. Survival data was also limited and therefore the rate from the US has been used. As noted before, the US has the highest health care expenditure per capita in the world [31], whereas Brazil spends nearly 8 times less than the US [31]. By using the figure from the US, we have probably overestimated survival for Brazil and depicted more optimistic outcomes. The analysis highlights the urgent need for better surveillance data in Brazil. Given the supposed overestimation of simulation, the forecasts for the real population would be expected to be even worse in terms of diseases burden. More accurate data will allow for more precise estimated in future work.

Brazil specific RR data was not accessible; therefore we have used the data for the European populations. Arguably, using a country specific RRs could change the picture, however, there is no evidence for Brazilian population RRs and we believe it is acceptable to use the data from Europe.

The data on total health care costs was also limited to a few diseases. Some cancer costs have not been found and therefore excluded from the projections. By including other disease costs, economic burden of obesity would be even higher. Though we have obtained costs of CHD, stroke, hypertension, diabetes and breast and colorectal cancers, the calculation methods were different among them. The CHD costs have been aggregated by summing up costs of IHD and MI costs and came from the same source as stroke (32). However, the costs are likely to be underestimated since they are only hospitalization costs and only among adults of age 20–60 [32]. Projected costs of CHD and stroke, therefore, are very low for a county of its size and by using these inputs we could show the potential growth of the costs due to increased obesity rates. More precise costs should be used in future calculations when data become available.

We usually model type 2 diabetes, however, the costs used are for diabetes (direct cost of type 1 and type 2). This would not have a substantial impact on calculations, since type 1 diabetes only accounts for 5–10% of all diabetes diagnosed [42].

The economic burden of breast and colorectal cancers are also likely to be underestimated, since the input costs were only the costs of the new cases. Despite these limitations, cancers impose considerable monetary constrains on health care system. In the model, we only included medical costs; non-medical costs, such as productivity loss, have not been incorporated, and would substantially increase already high cost. Moreover, we have simulated only 13 diseases; other obesity-related conditions such as infertility [43] were not modelled which is likely to increase health care burden.

For this project, only adult BMI data has been used. Incorporating childhood trends could alter our projections. As is common, data on BMI is limited to self-reported surveys and, as noted elsewhere [44], individuals tend to underreport their weight. Using anthropometric data, rather than self-reported BMI data (as people tend to underreport their weight [45]) would increase the estimates. In addition, unlike the 1989 and 2003 data, which were nationally representative, the 2006–2010 VIGITEL data were only for cities. Using only urban data for BMI prevalence for the later years probably inflated the obesity growth, since urban areas in low and middle income nations experience growth in obesity before rural areas [46].

## Conclusion

Our analysis shows that the percentage of overweight and obesity and the associated disease and economic burden will substantially increase in Brazil. The increase will be higher among men than among women. Arguably, lower rates of obesity among women can be linked to an increased underreporting from females [45]. Importantly, decrease has been observed in the past among females of urban areas of Brazil [17]; [18] and BMI projections for this paper are drawn from the data from urban region of Brazil. This is also likely to have influenced presented obesity levels among women. Further research is needed to examine the possible reasons for gender differences.

The obesity increase is very likely to be linked to increased consumption of ultra-processed food which is a source of cheaper calories, sedentary lifestyle and/or economic transition. In turn, high obesity rates are drivers of increased NCDs morbidity rates, as well as higher costs for the healthcare system. Our analysis shows that even relatively small reductions in the level of BMI can lead to a substantial disease in the reduction and cost savings for the health care system. These results also can be translated to average costs per case and can be used in cost-effectiveness analysis for comparing programs and policies affecting obesity in Brazil.

Projected high burden of disease shows that a crucial change in policies is needed to tackle and avoid high rates of diseases and health care costs. Due to increased prevalence rates in weight gain and sedentary behaviour, some steps have been made in late 90 s. In 1996, with the financial support from the Health Secretariat of the state of Sao Paulo, the physical activity program – Agita, the program promoting adoption of physical activity was launched. As a result, physical activity among students, workers and the elderly increased, and the program has been considered as a model for other developing countries by the WHO [47]. The food marketing restrictions have been also advocated by various agencies, however it was regarded as ‘unconstitutional’ (pg.31) and did not come into force [48].

Our model shows that a 1% reduction in the mean BMI alone can lead to a large change in NCD rates. These attenuated trends can be accomplished by either prevention (of weight gain) or weight reduction interventions across the whole population. The OECD has assessed some prevention programmes for Mexico and concluded that they are all cost-effective, some with an immediate effectiveness and some over the long term [49] and similar method could be applied to Brazil. Gortmaker and colleagues [50] recommend multiple policy recommendations for preventing and controlling obesity that may also be applicable to countries such as Brazil. A 5% reduction will be more difficult to accomplish, however, we believe it is achievable with thorough planning and stronger policy. It would require different stakeholders to work along and strong leadership from health care community and government willingness to tackle high rates of morbidity and NCD mortality in Brazil. A more comprehensive policy approach, better surveillance data on health care resources and continued evaluation of policies will be needed to ensure their success in Brazil.

Several limitations have been drawn in the previous section. As noted, multiple disease costs have not been found, therefore incorporating them into the model, along with other conditions (except these 13 diseases) will, however, increase economic constrains. Economic burden is likely to increase by incorporating non-medical costs. Lack of measured BMI data, outdated disease incidence rates, absence of healthcare costs for multiple conditions, as well as utilization of survival figures from other countries can lead to inaccuracy in presenting the projected data. However, we believe that our projections are timely and draw attention of the policy makers to important issues rising in Brazil. Our approach is conservative, and incorporating more comprehensive data will more likely increase projected disease burden.

## Supporting Information

File S1 Figure S1–S24 Distribution of the BMI (kg/m^2^) among males and females aged 20–75+.
(DOC)

File S2 Table S2 Cost input in the model.
(DOC)

File S3 Table S3 Cumulative incidence cases by intervention.
(DOC)
