# Supplementary material for: Health and Economic Burden of Obesity in Brazil
Source: PLoS One. 2013 Jul 11;8(7):e68785. doi: 10.1371/journal.pone.0068785 (PMC3708916; doi:10.1371/journal.pone.0068785)
Supplement: File S1 — Figure S1–S24 Distribution of the BMI (kg/m2) among males and females aged 20–75+. (DOC) [file pone.0068785.s001.doc]

**Health and economic burden of obesity in Brazil**

**Supporting Information**

Figure S1 Distribution of the BMI (kg/m²) among males aged 20-24


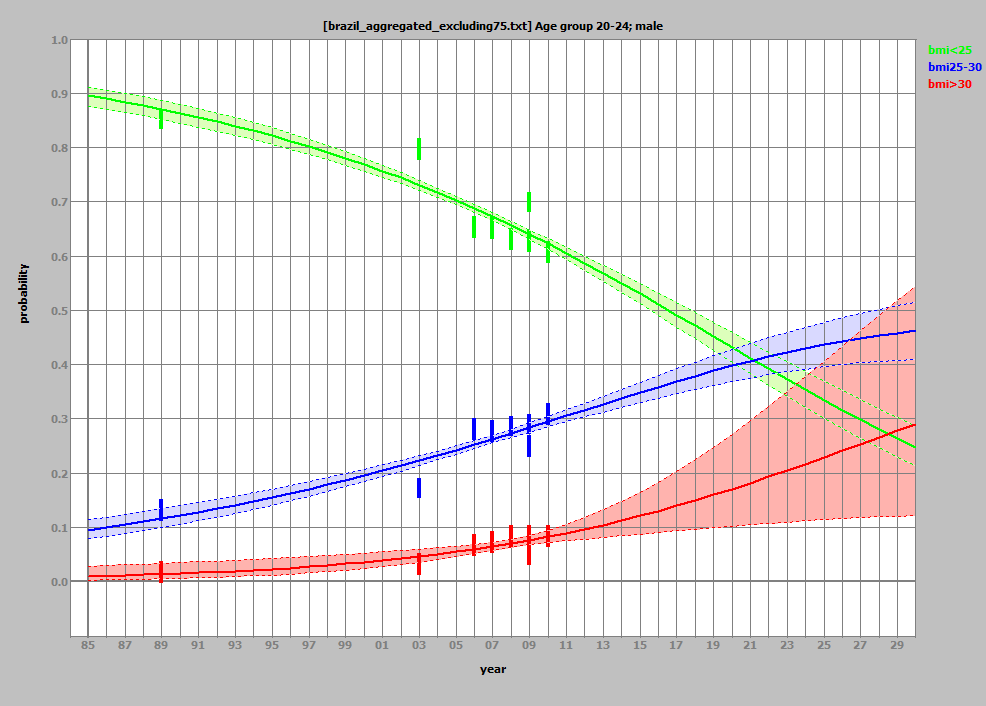


Figure S2 Distribution of the BMI (kg/m²) among females aged 20-24


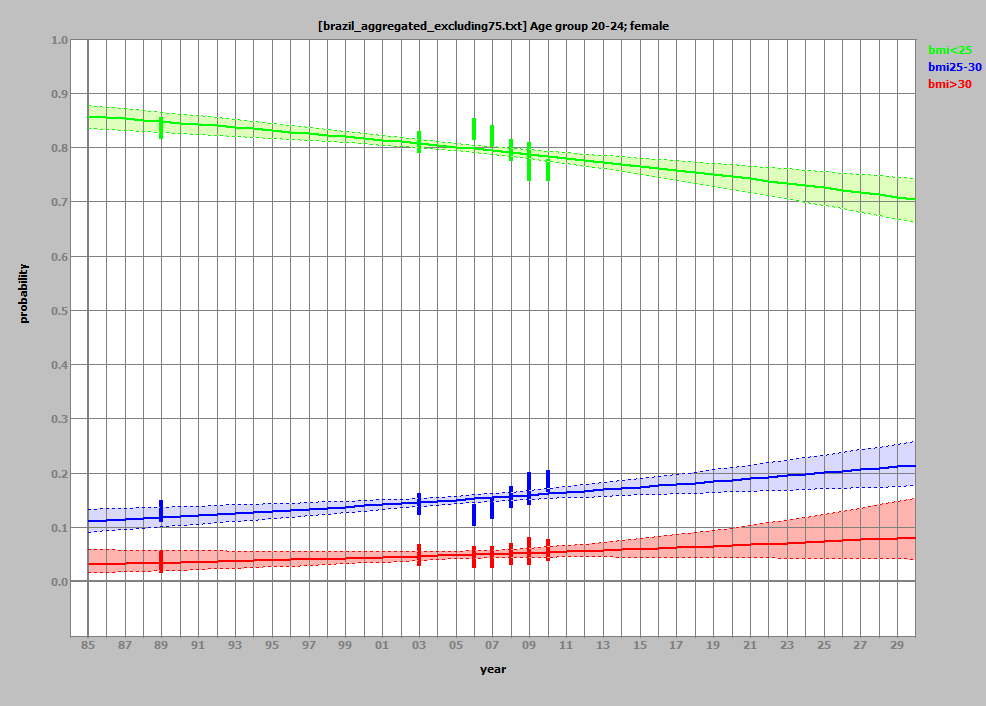


**Figure S3 Distribution of the BMI (kg/m²) among males aged 25-29**

**
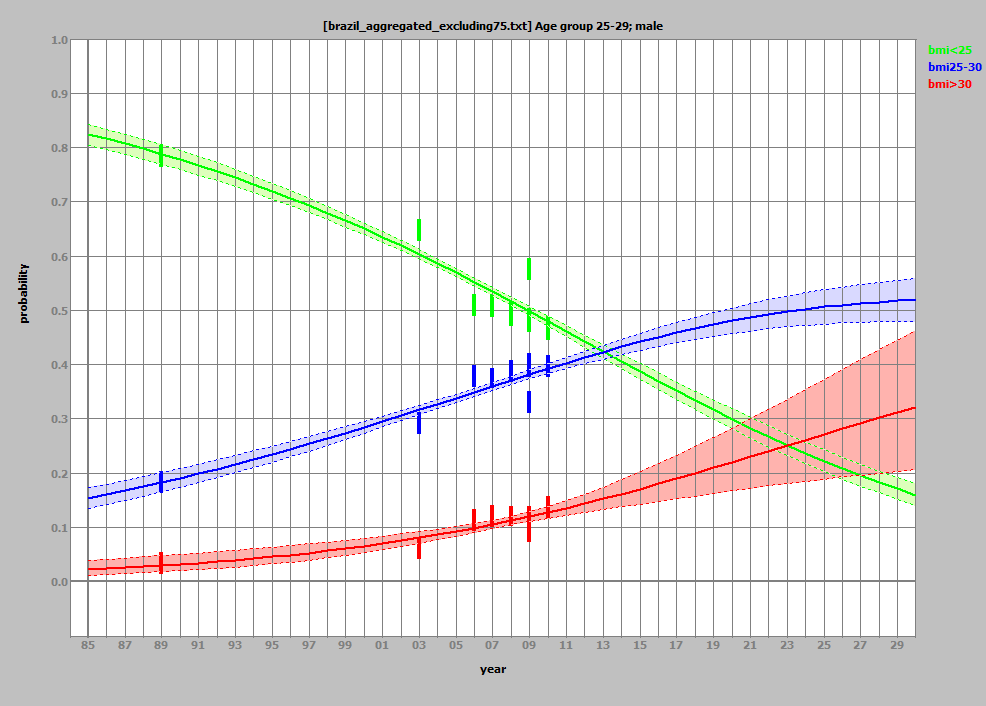
**

Figure S4 Distribution of the BMI (kg/m²) among females aged 25-29


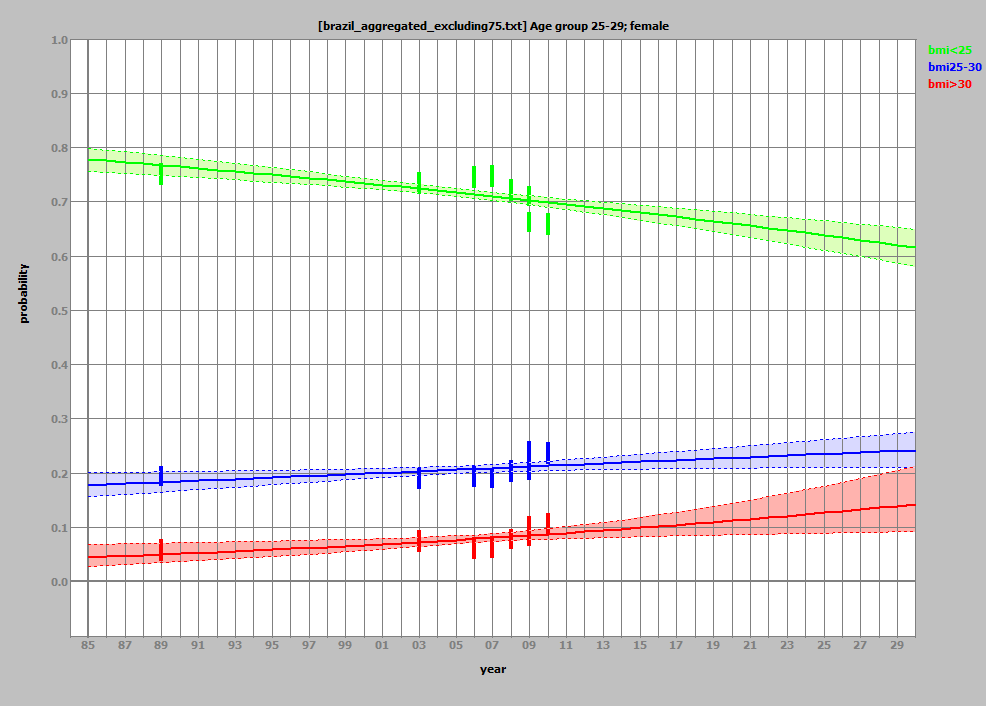


Figure S5 Distribution of the BMI (kg/m²) among males aged 30-34


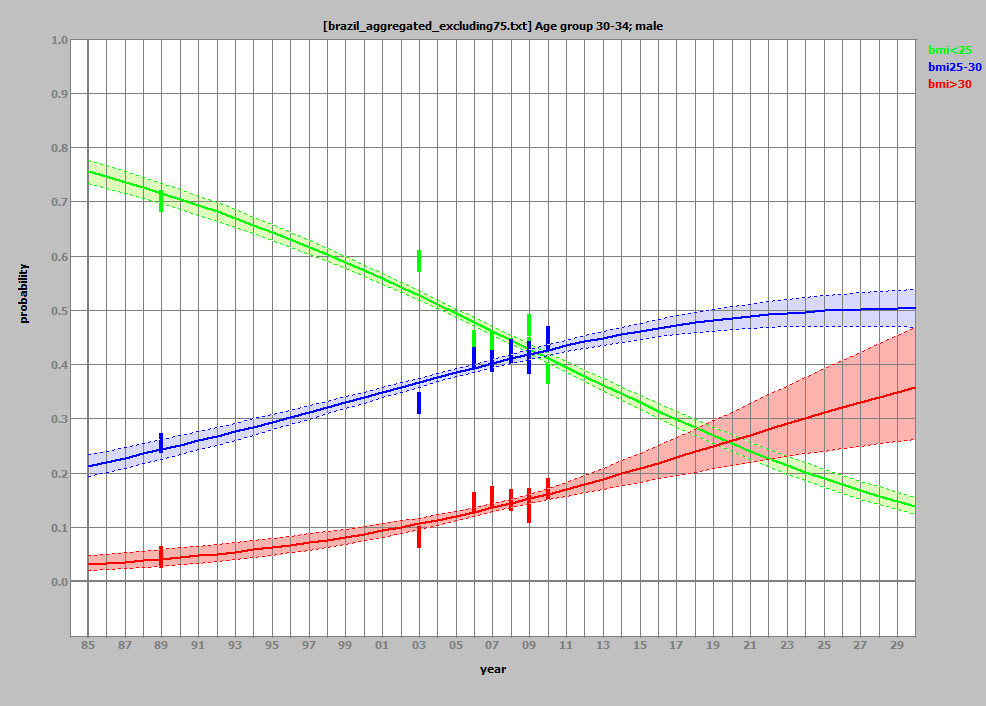


Figure S6 Distribution of the BMI (kg/m²) among females aged 30-34


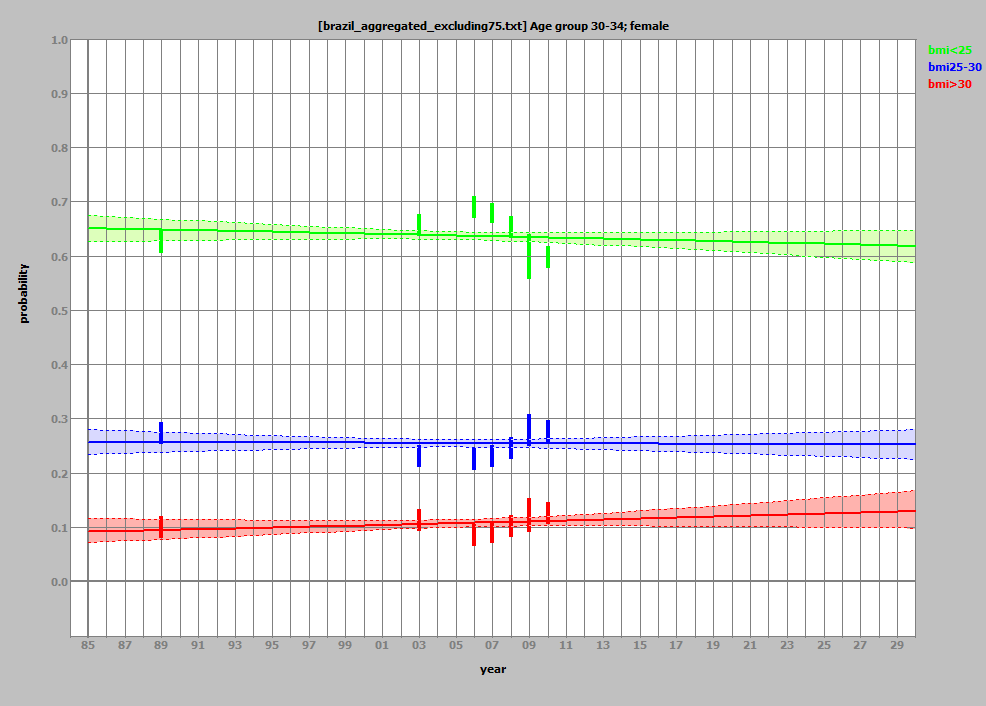


Figure S7 Distribution of the BMI (kg/m²) among males aged 35-39


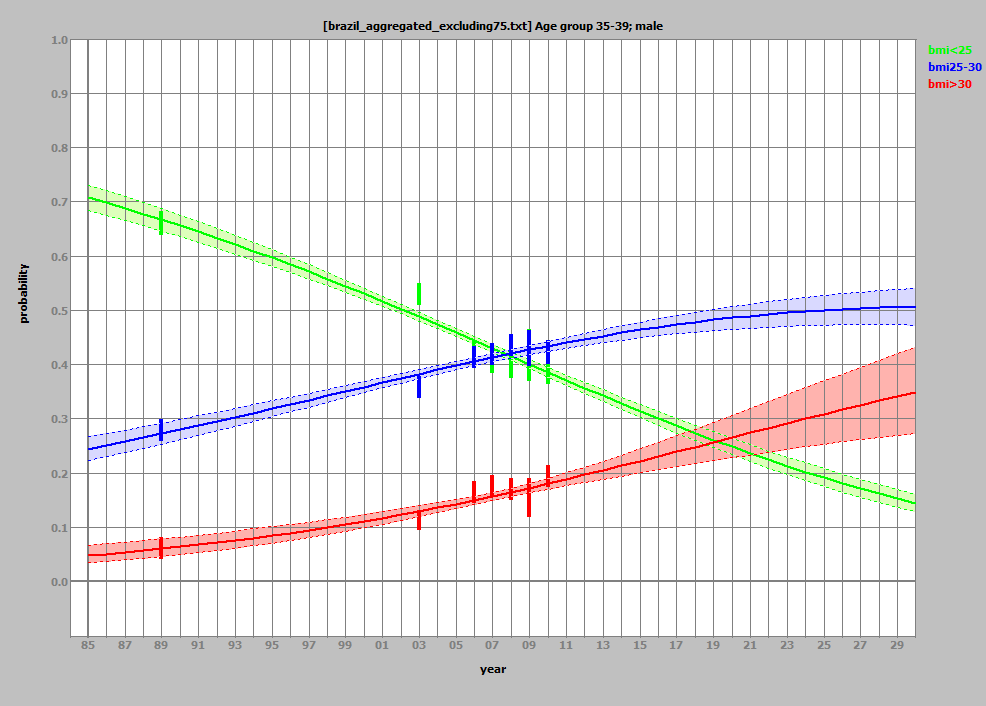


Figure S8 Distribution of the BMI (kg/m²) among females aged 35-39


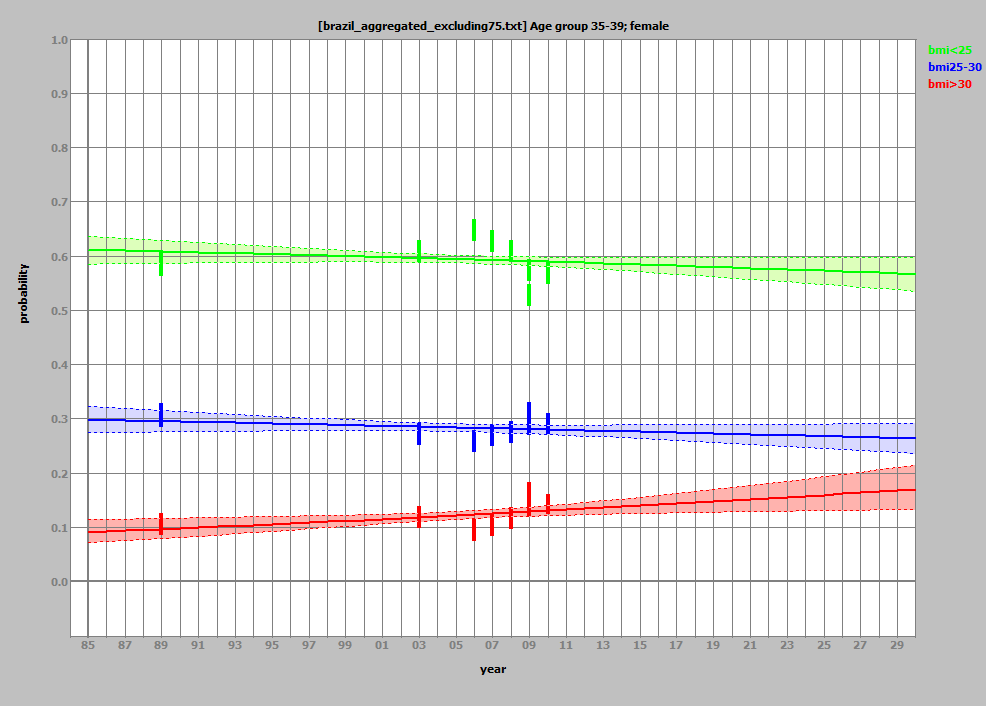


Figure S9 Distribution of the BMI (kg/m²) among males aged 40-44


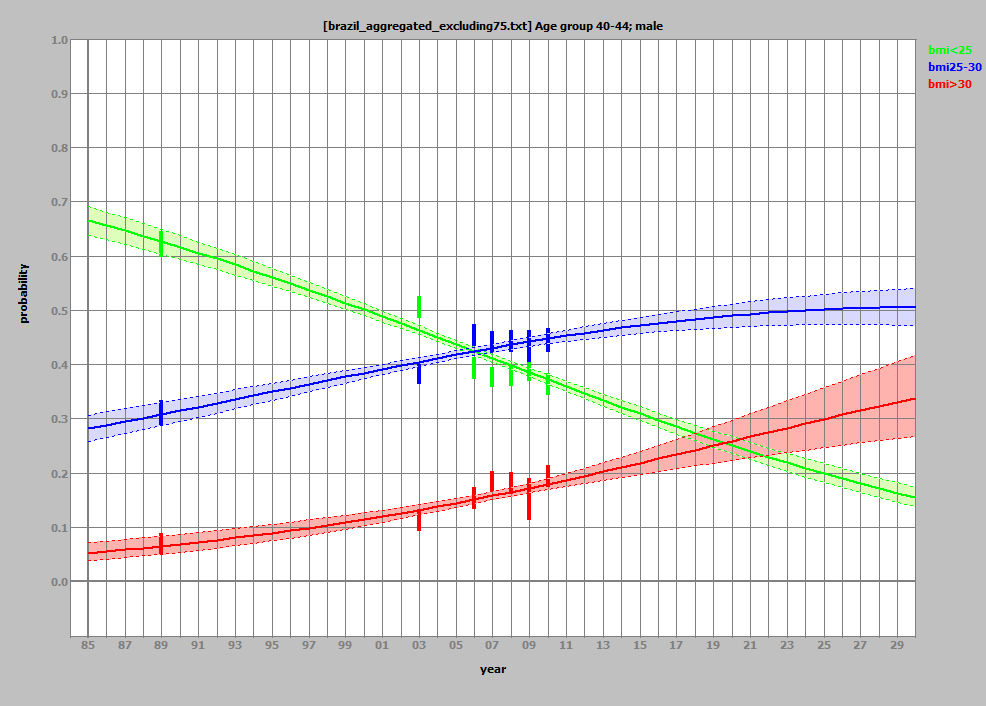


Figure S10 Distribution of the BMI (kg/m²) among females aged 40-44

**
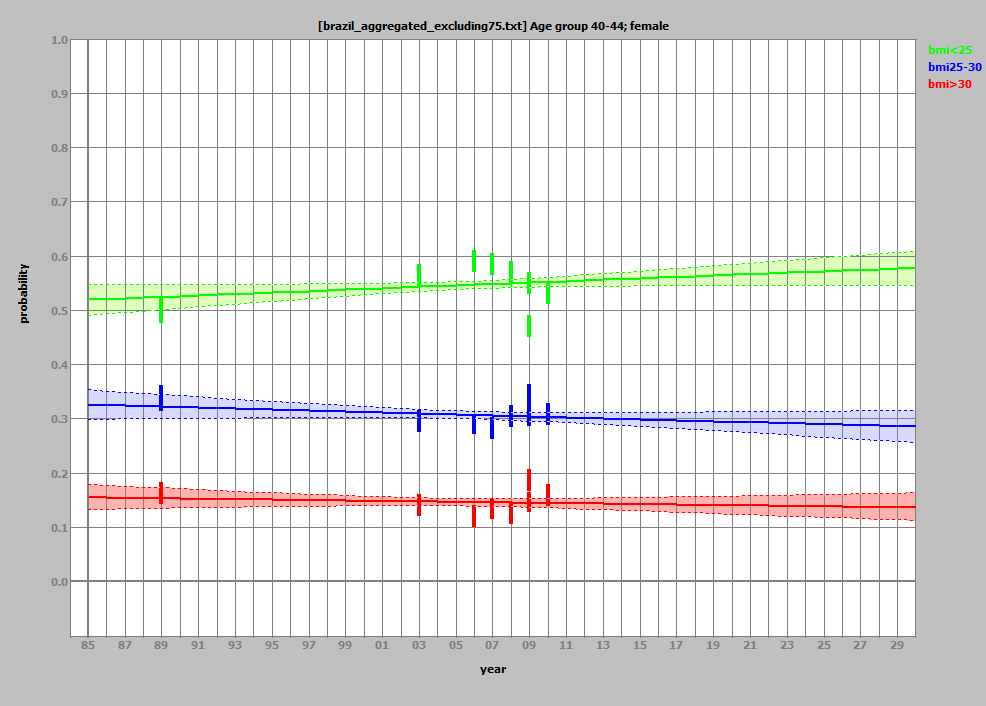
**

Figure S11 Distribution of the BMI (kg/m²) among males aged 45-49

**
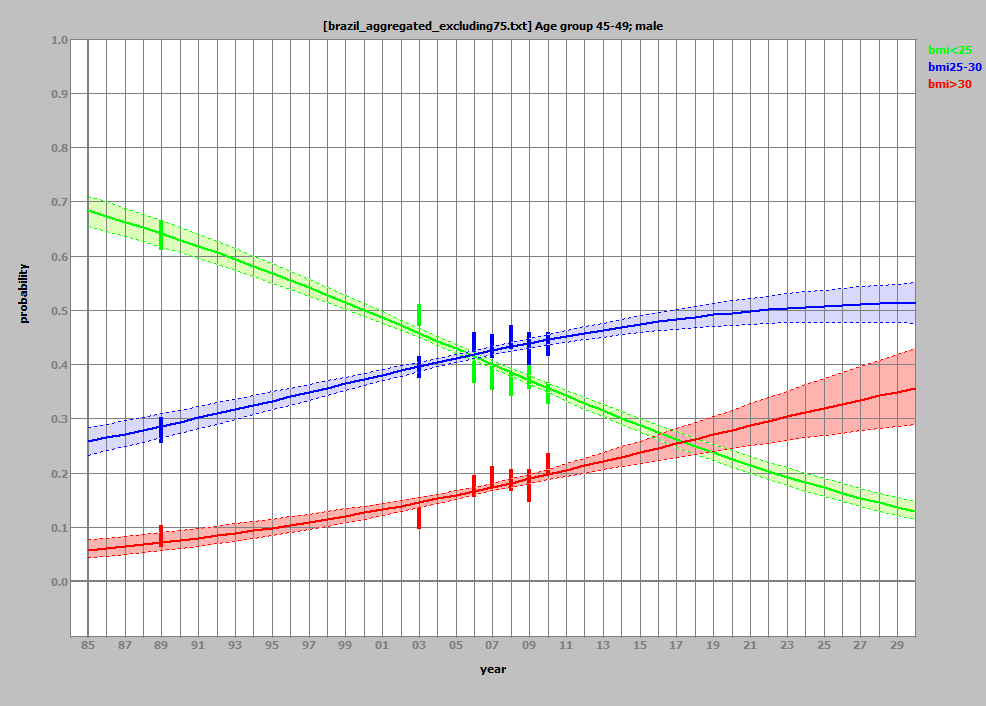
**

Figure S12 Distribution of the BMI (kg/m²) among females aged 45-49


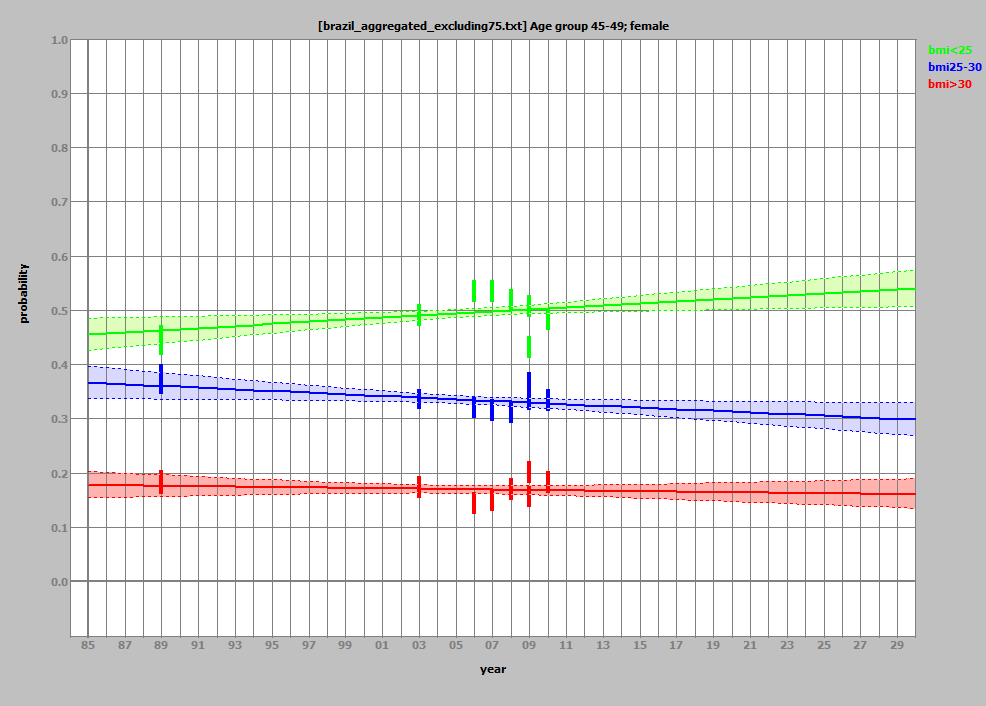


Figure S13 Distribution of the BMI (kg/m²) among males aged 50-54


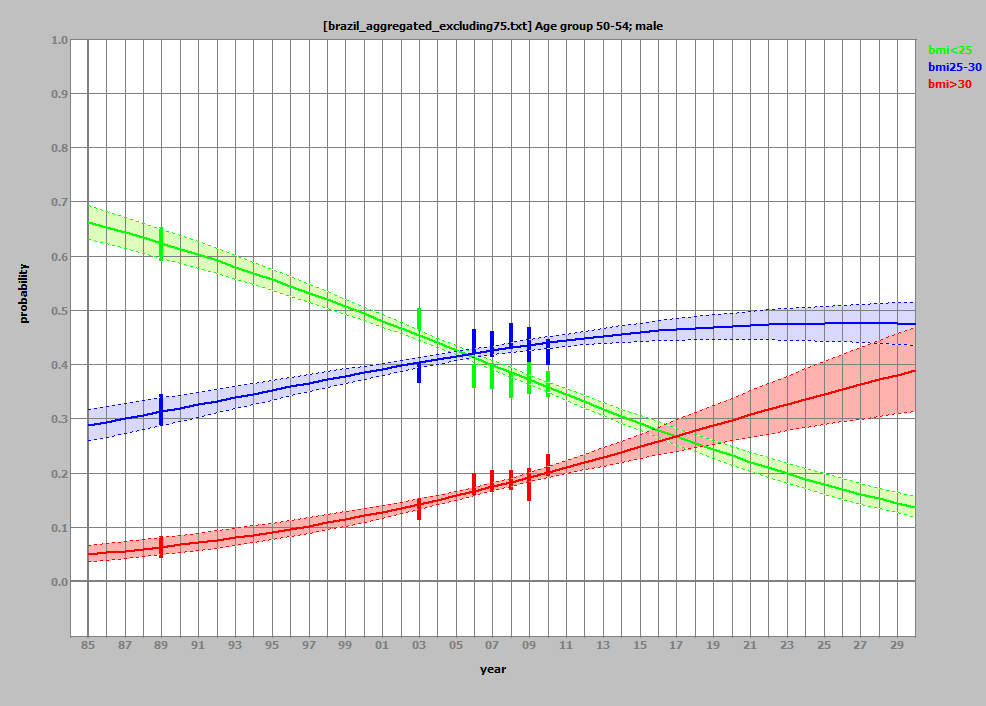


Figure S14 Distribution of the BMI (kg/m²) among females aged 50-54

**
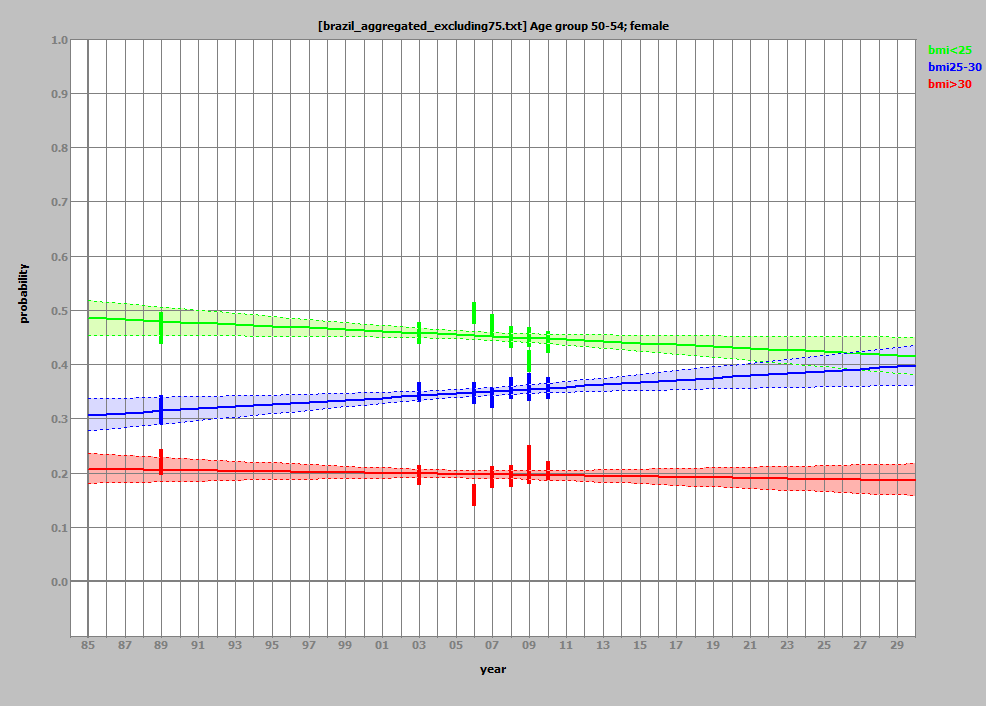
**

Figure S15 Distribution of the BMI (kg/m²) among males aged 55-59

**
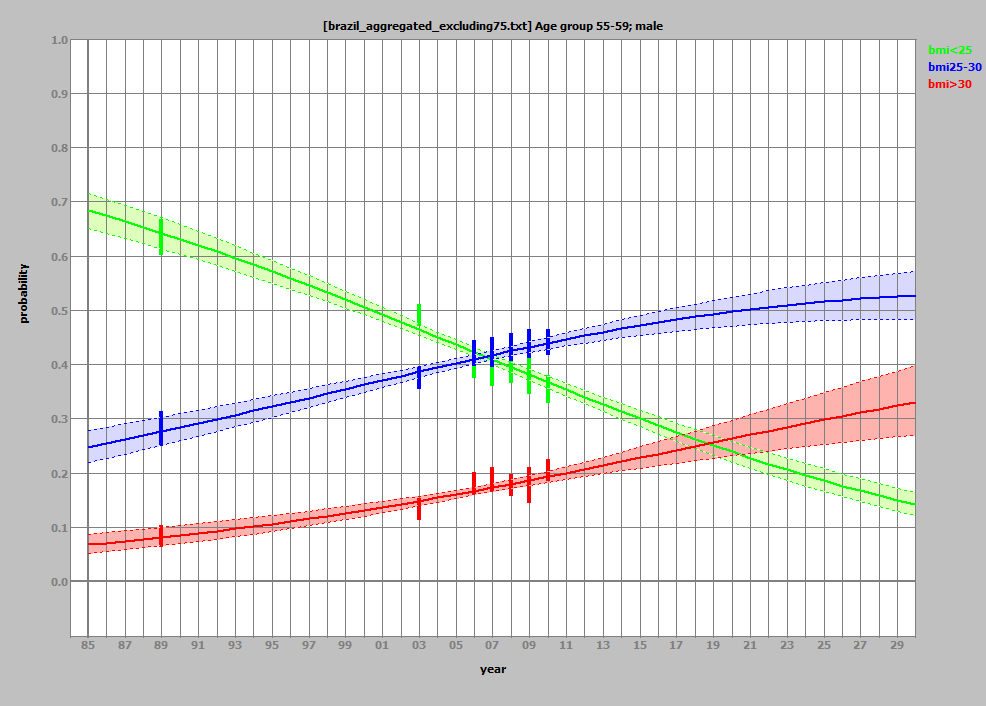
**

Figure S16 Distribution of the BMI (kg/m²) among females aged 55-59


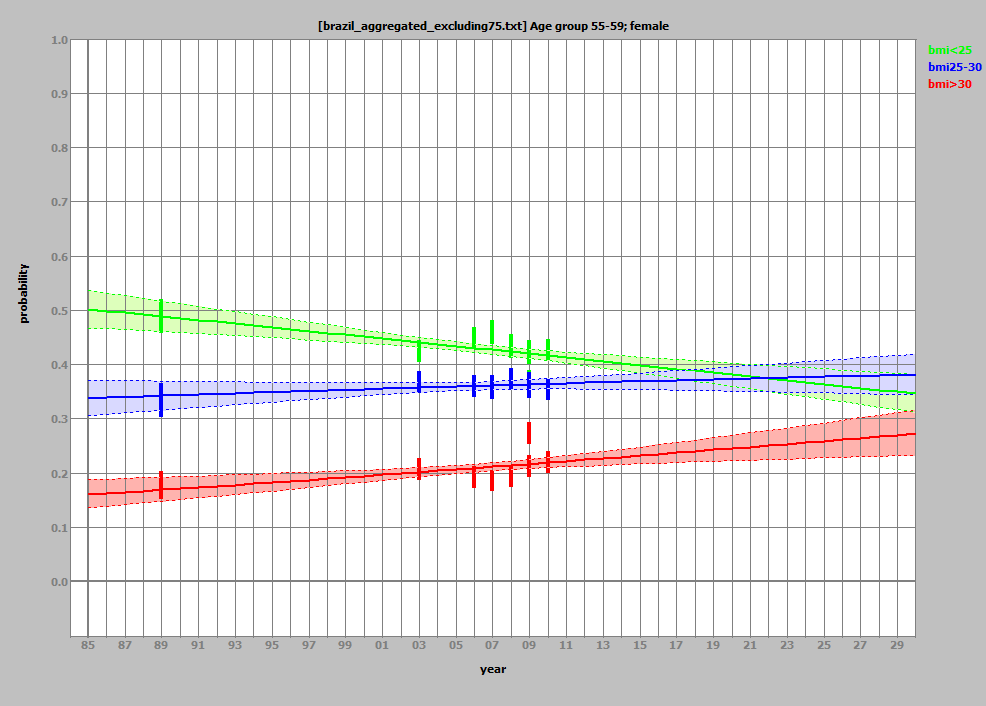


Figure S17 Distribution of the BMI (kg/m²) among males aged 60-64

**
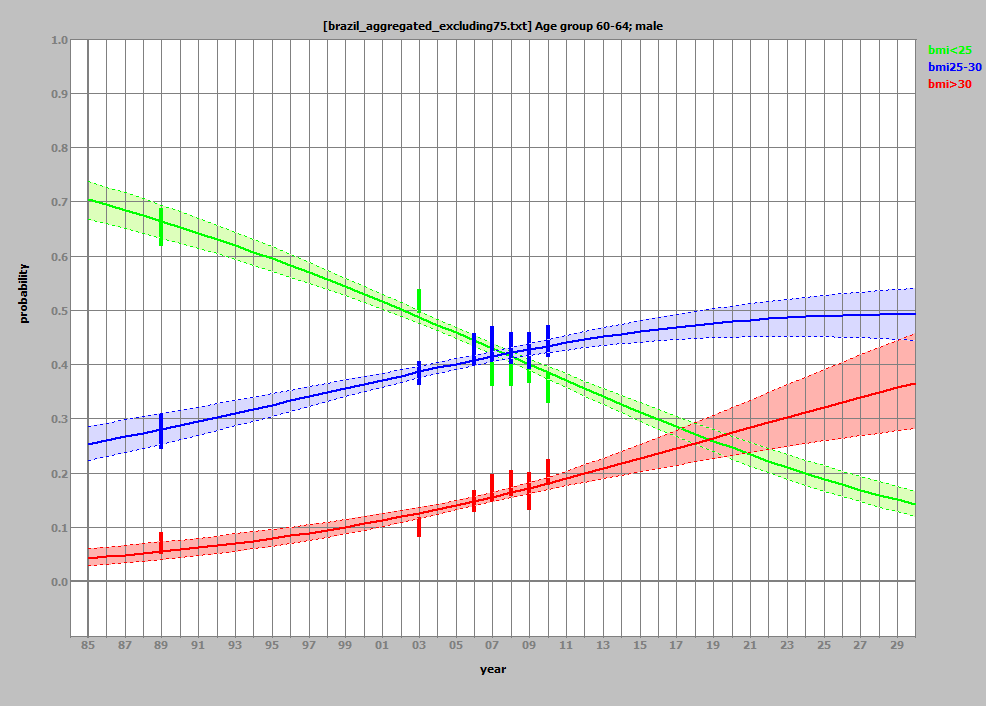
**

Figure S18 Distribution of the BMI (kg/m²) among females aged 60-64


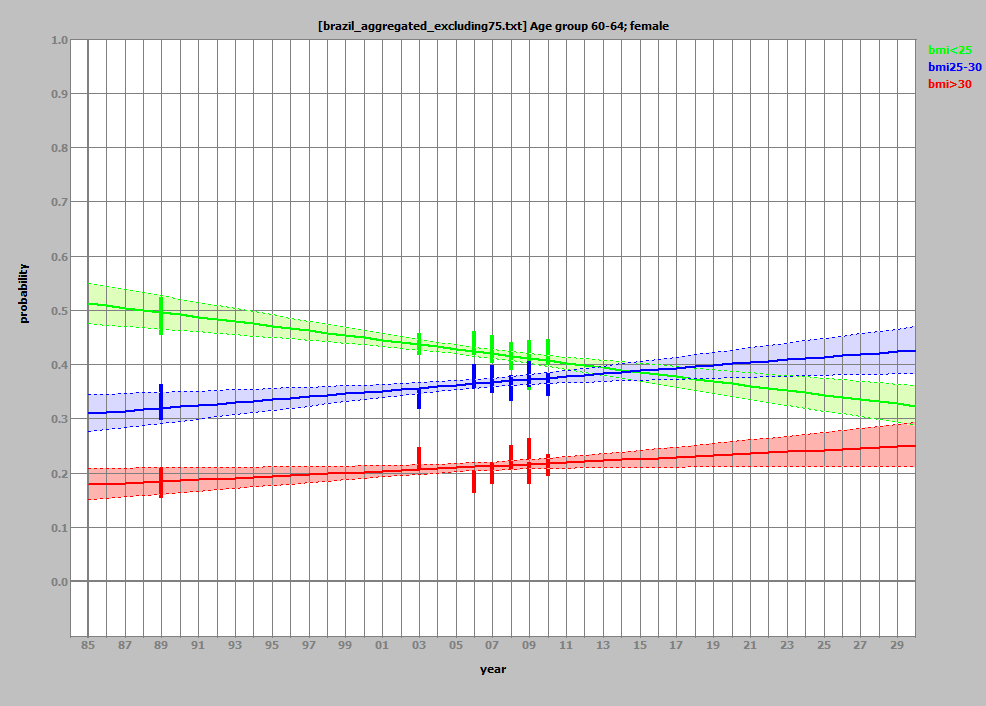


Figure S19 Distribution of the BMI (kg/m²) among males aged 65-69

**
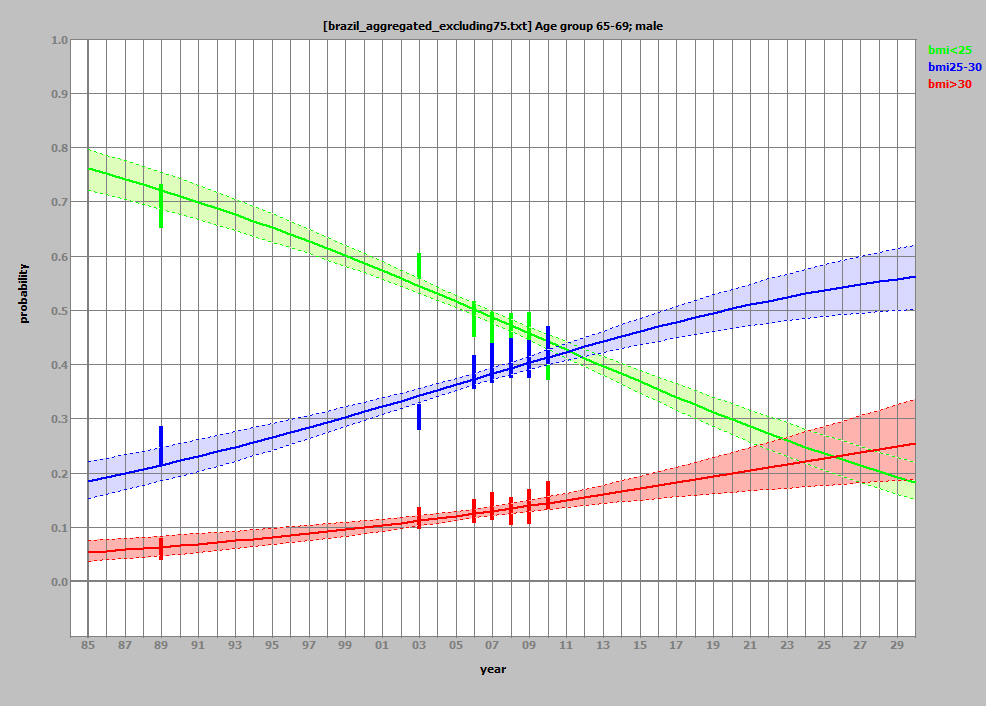
**

Figure S20 Distribution of the BMI (kg/m²) among females aged 65-69

**
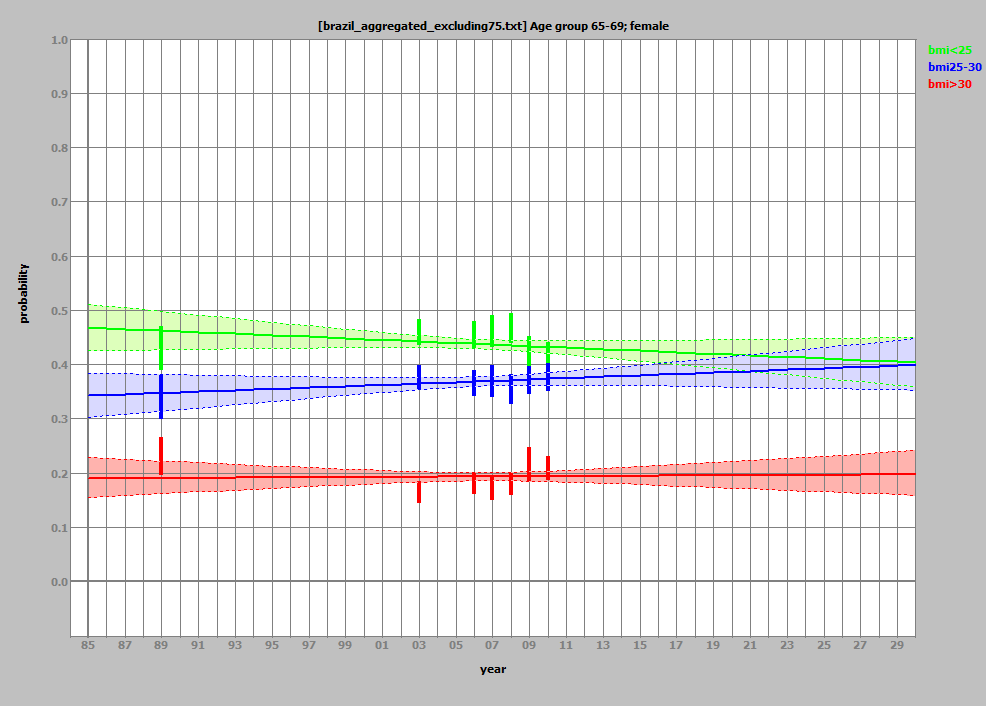
**

Figure S21 Distribution of the BMI (kg/m²) among males aged 70-74


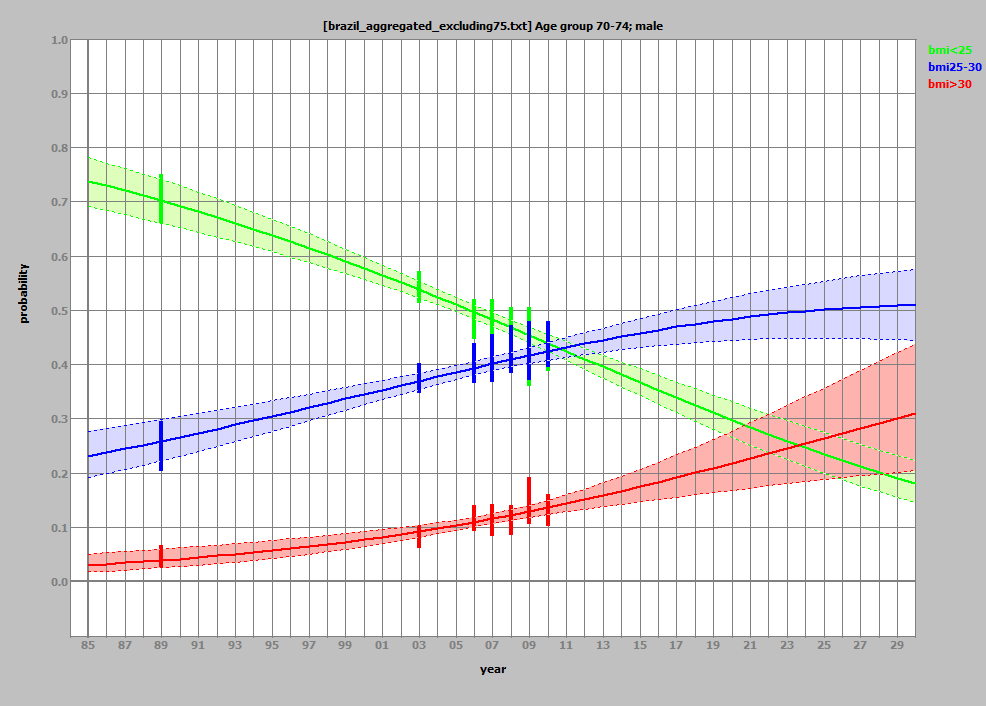


Figure S22 Distribution of the BMI (kg/m²) among females aged 70-74


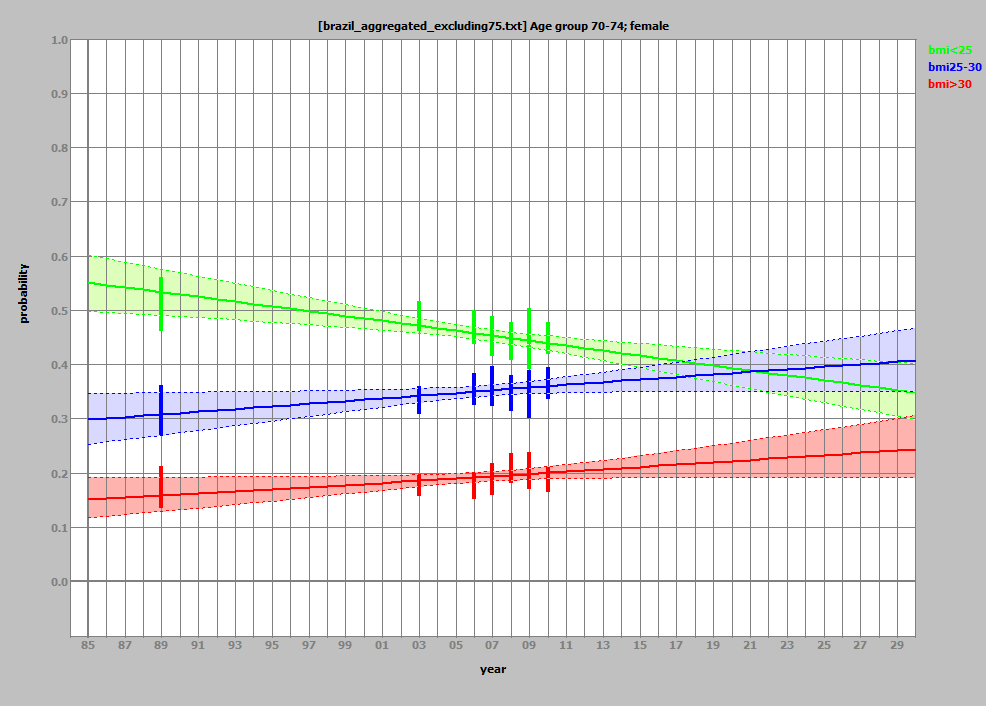


Figure S23 Distribution of the BMI (kg/m²) among males aged 75+

**
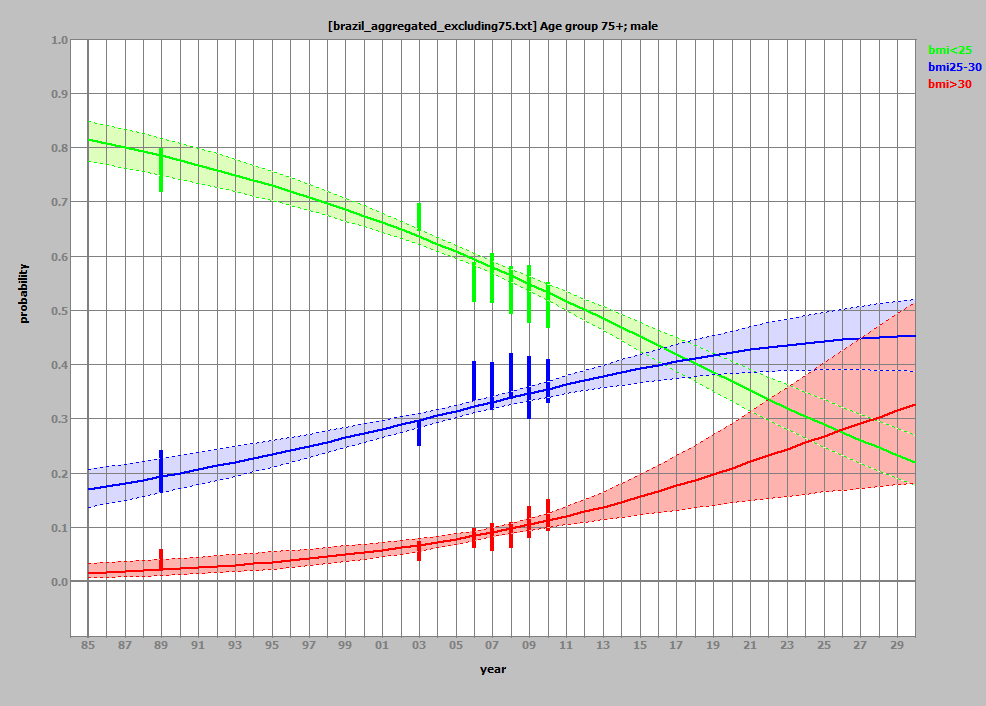
**

Figure S24 Distribution of the BMI (kg/m²) among females aged 75+

**
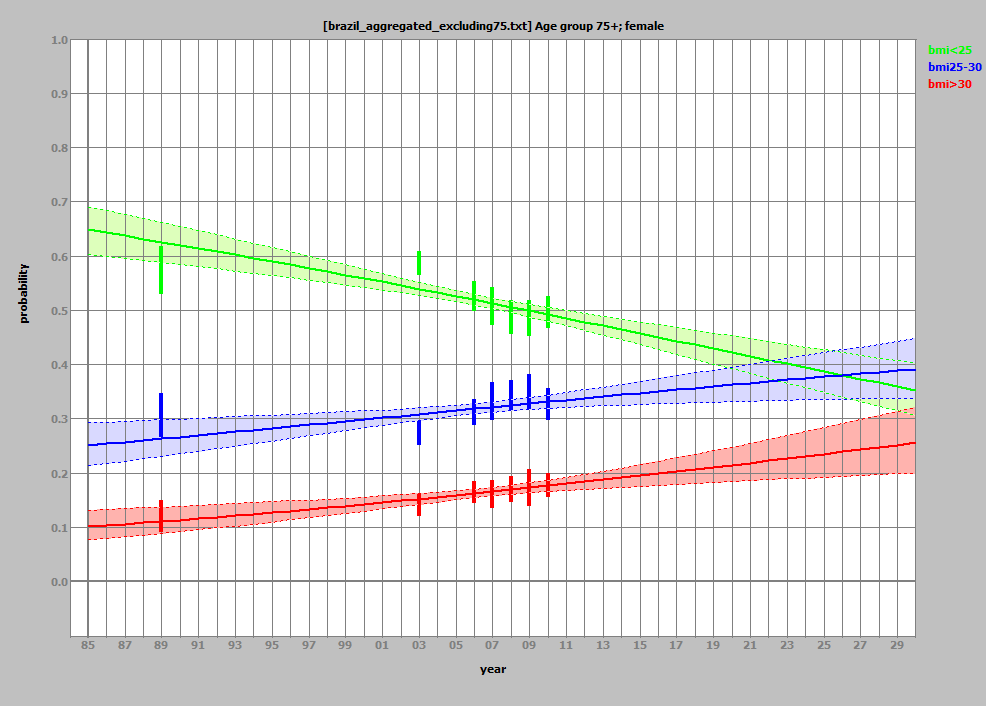
**
