# Supplementary material for: Health and Economic Burden of Obesity in Brazil
Source: PLoS One. 2013 Jul 11;8(7):e68785. doi: 10.1371/journal.pone.0068785 (PMC3708916; doi:10.1371/journal.pone.0068785)
Supplement: File S2 — Table S2 Cost input in the model. (DOC) [file pone.0068785.s002.doc]

| **Disease** | **Cost per year** | **Year and Source** | **Cost in 2010** | **Annual rate in inflation change** | **Cost ingredients** | **Cost perspective** |
| --- | --- | --- | --- | --- | --- | --- |
| **Diabetes** | $3,952,300,000.00 | 2000 (34) | $5,004,754,800.00 | 27% | Direct Cost (drugs, hospitalizations, consultations and management of complications) | N/A |
| **Hypertension** | $398,900,000.00 | 2005 (35) | $445,377,058.00 | 12% | Direct cost (treatment of Systemic arterial hypertension) | Brazilian Unified Healthcare System (Sistema Único de Saúde, SUS) |
| **MI** | $9,490,505.00 | 2001 (32) |  | | Direct cost (hospitalization) | Brazilian Unified Healthcare System (Sistema Único de Saúde, SUS) |
| **Other IHD** | $53,293,230.00 | 2001 (32) |  |  | Direct cost (hospitalization) | Brazilian Unified Healthcare System (Sistema Único de Saúde, SUS) |
| **MI & other IHD (used as CHD)** | $62,783,735.00 | 2001 (32) | $81,297,162.00 | 23% | From the above | Brazilian Unified Healthcare System (Sistema Único de Saúde, SUS) |
| **Stroke** | $6,829,937.00 | 2001 (32) | $88,432,922.00 | 23% | Direct cost (hospitalization) | Brazilian Unified Healthcare System (Sistema Único de Saúde, SUS) |
| **Breast Cancer** | $157,364,603.00 | 2009 (33) | $159,945,084.00 | 2% | Direct costs (medical plus non-medical) and productivity losses deriving from new cancer cases | N/A |
| **Colorectal Cancer** | $112,786,011.00 | 2009 (33) | $114,635,488.00 | 2% | Direct costs (medical plus non-medical) and productivity losses deriving from new cancer cases | N/A |

**Supporting Information**

**Table S2** **Cost input in the model**

*Costs of MI and IHD have been summed up to be used in the model and then have been inflated to 2010 [36].
