# Supplementary material for: Health and Economic Burden of Obesity in Brazil
Source: PLoS One. 2013 Jul 11;8(7):e68785. doi: 10.1371/journal.pone.0068785 (PMC3708916; doi:10.1371/journal.pone.0068785)
Supplement: File S3 — Table S3 Cumulative incidence cases by intervention. (DOC) [file pone.0068785.s003.doc]

**Supporting Information**

*Intervention 0 – BMI projections according to the past trend*

*Intervention 1 – 1% reduction in BMI across the population in 2010*

*Intervention 2 – 5% reduction in BMI across the population in 2010*

Table S3 Cumulative incidence cases by intervention. Numbers in square brackets represent standard deviation.

| **Cumulative incidence cases for all adults (per 100,000)** | | |  |  |  |  |
| --- | --- | --- | --- | --- | --- | --- |
|  |  |  |  |  |  |  |
|  | **Year** | **All 8 Cancers** | **CHD & Stroke** | **Knee Osteoarthritis** | **Hypertension** | **Type 2 Diabetes** |
| **Intervention 0** | 2010 | 58 [±2] | 284 [±5] | 599 [±7] | 879 [±8] | 172 [±4] |
|  | 2020 | 696 [±7] | 3,361 [±16] | 6,953 [±23] | 9,969 [±27] | 2,006 [±12] |
|  | 2030 | 1,475 [±10] | 7,154 [±23] | 14,105 [±32] | 19,593 [±38] | 4,188 [±17] |
|  | 2040 | 2,447 [±13] | 12,072 [±30] | 22,154 [±40] | 29,668 [±46] | 6,756 [±22] |
|  | 2050 | 3,635 [±16] | 18,367 [±37] | 31,220 [±49] | 40,560 [±55] | 9,744 [±27] |
| **Intervention 1** | 2010 | 58 [±2] | 283 [±5] | 584 [±7] | 856 [±8] | 158 [±4] |
|  | 2020 | 688 [±7] | 3,296 [±16] | 6,805 [±23] | 9,610 [±27] | 1,888 [±12] |
|  | 2030 | 1,458 [±10] | 6,994 [±23] | 13,792 [±32] | 18,932 [±37] | 3,912 [±17] |
|  | 2040 | 2,415 [±13] | 11,792 [±29] | 21,692 [±40] | 28,730 [±46] | 6,238 [±21] |
|  | 2050 | 3,582 [±16] | 17,870 [±37] | 30,537 [±48] | 39,358 [±54] | 8,926 [±26] |
| **Intervention 2** | 2010 | 55 [±2] | 270 [±5] | 542 [±7] | 794 [±8] | 134 [±3] |
|  | 2020 | 672 [±7] | 3,148 [±15] | 6,403 [±22] | 9,024 [±26] | 1,593 [±11] |
|  | 2030 | 1,416 [±10] | 6,658 [±22] | 13,089 [±31] | 17,711 [±36] | 3,363 [±16] |
|  | 2040 | 2,341 [±13] | 11,203 [±28] | 20,607 [±39] | 26,814 [±44] | 5,379 [±20] |
|  | 2050 | 3,462 [±16] | 16,944 [±36] | 29,012 [±47] | 36,637 [±52] | 7,664 [±24] |
|  |  |  |  |  |  |  |
